# Complete AV Block in Vaccinated COVID-19 Patient

**DOI:** 10.1155/2022/9371818

**Published:** 2022-03-30

**Authors:** Kevin Lee, Osman Rahimi, Neelesh Gupta, Chowdhury Ahsan

**Affiliations:** ^1^Department of Internal Medicine, University of Nevada, Las Vegas, 1707 W Charleston Blvd, Suite 100, Las Vegas, NV 89102, USA; ^2^Department of Cardiology, University of Nevada, Las Vegas, 1800 W Charleston Blvd, Las Vegas, NV 89102, USA

## Abstract

*Background*. Coronavirus 2019 (COVID-19) was initially identified approximately in December 2019 at Wuhan, China, as patients presented with vague prodromal and respiratory symptoms. With the developing investigation of its clinical manifestation, cardiac symptoms have been widely reported including acute coronary syndromes, myocarditis, arrhythmias, heart failure, and cardiac arrest. *Case Summary*. An 84 year-old male with history of coronary artery disease, hypertension, and hyperlipidemia presented to an outside urgent care with prodromal symptoms. The patient had received the second Pfizer vaccine three months prior. This presentation, he was found to be COVID-19 positive as well as bradycardic with a complete AV block. He was transferred to a tertiary center for further evaluation and management. However, after transfer, the patient refused further invasive cardiac interventions and after medical therapy was discharged home in complete AV block. *Discussion*. We report a novel case of a Pfizer-vaccinated patient whose initial presenting symptoms of COVID-19 included a complete AV block as well as the challenges and difficulties in approaching such patients. Although this patient's etiology of his complete AV block may result from multiple factors, given the acuity in setting of concurrent COVID-19 infections, top differentials include viral myocarditis, COVID-19-induced Takotsubo cardiomyopathy complicated by a complete AV-block, or a direct conduction pathway infection. Management of patients should focus on a multidisciplinary approach, and prevention is critical via vaccination.

## 1. Introduction

Severe acute respiratory syndrome coronavirus-2 (SARS-CoV-2) also known as coronavirus 2019 (COVID-19) was initially identified approximately in December 2019 at Wuhan, China, as patients presented with vague prodromal and respiratory symptoms. The virus rapidly progressed to a pandemic with surmountable casualties. With the developing investigation of its clinical manifestation, cardiac symptoms have been widely reported including acute coronary syndromes, myocarditis, arrhythmias, heart failure, and cardiac arrest [1].

We report a novel case of a Pfizer-vaccinated SARS-CoV-2 positive patient who presented with a complete AV block. Although previous high-degree AV blocks have been reported as a later complication of COVID-19 [2–4], this is the first documented case whose initial presenting symptom was a complete AV block in a vaccinated patient.

## 2. Case Presentation

An 84-year-old male with a history of coronary artery disease, hypertension, and hyperlipidemia presented to an urgent care in with body aches, malaise, fatigue, and dyspnea for three days. No other infectious symptoms were endorsed. At the outside facility, he was found to be bradycardic with a complete AV block. Total of atropine 1.0 mg was administered at that encounter. Afterwards, the patient went into a supraventricular tachycardia briefly with spontaneous return to a complete AV block. He was placed on transcutaneous pacing and transferred to our facility for further evaluation and management. Upon encounter, he denied any symptoms of lightheadedness, dizziness, palpitations, orthopnea, and chest pain. Patient also noted that he received both doses of the Pfizer vaccine at the beginning and end of April 2021, approximately six months prior to presentation. No booster doses were administered. Furthermore, he was recently seen by his cardiologist approximately a week prior with no abnormalities reported at the time. His previously known heart rate ranged between 77 and 99. Prior PR interval was 128. He had no history of tobacco, alcohol, or drug use. A possible culprit home medication was atenolol 50 mg daily which he reported taking as instructed. No other pertinent or new medications were noted in the patient's history.

**Figure 1:**
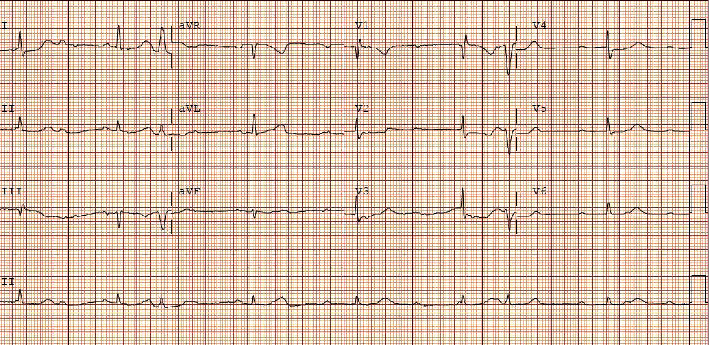
Initial presenting EKG.

**Figure 2:**
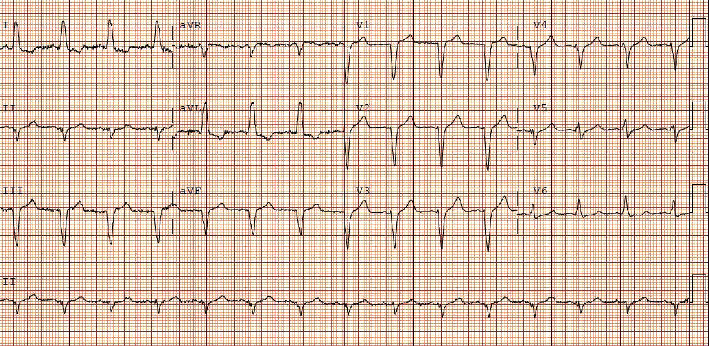
EKG 6 months prior to presentation.

**Figure 3:**
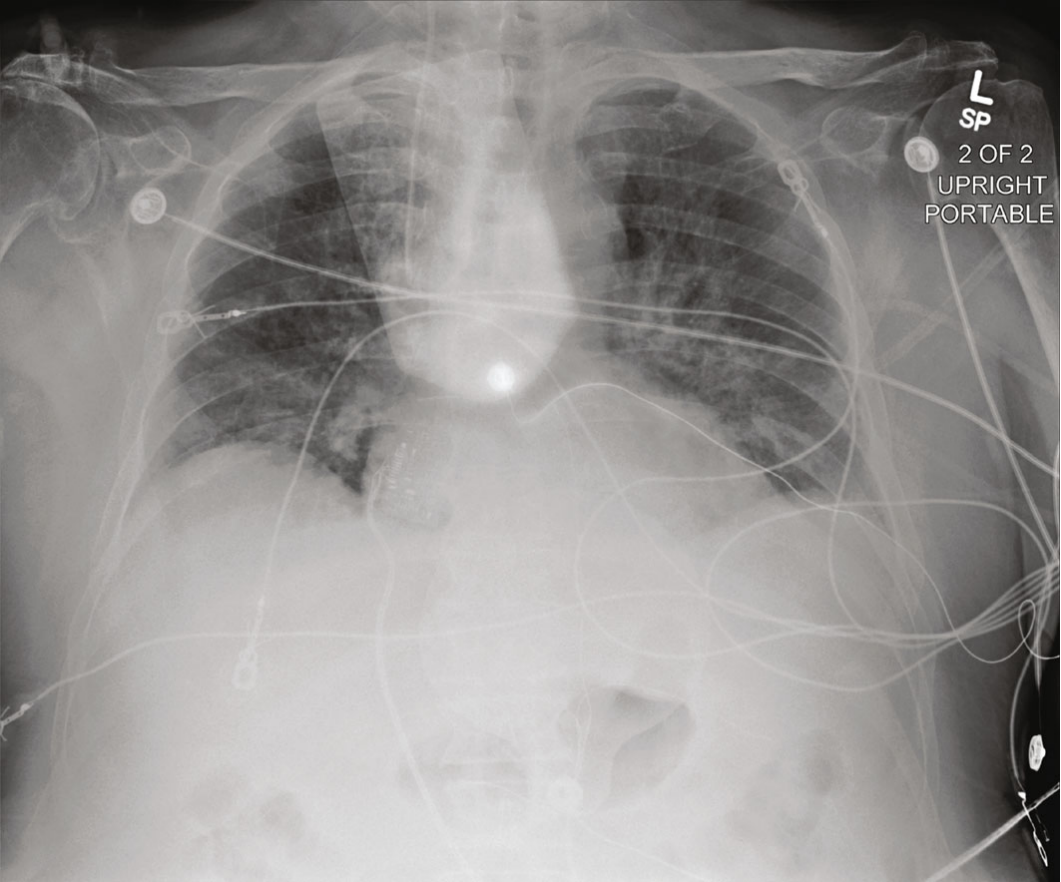
CXR on initial presentation.

On initial evaluation, he appeared comfortable and euvolemic. His blood pressure was 96/36 with an oxygen saturation of 82%. He was transcutaneously paced at a rate of 60 bpm at 70 mA. No audible bruits or murmurs were heard. The lungs were clear to auscultation. No peripheral edema was present. Underlying EKG without pacing showed an atrial rate of 128 bpm and a ventricular rate of 48 bpm (Figure 1). His previous EKG in May 2021 showed sinus rhythm with left bundle branch block and a prolonged QTc (Figure 2). Chest X-ray on admission showed pulmonary infiltrates without cardiomegaly (Figure 3). Initial troponin was 52 ng/L with a peak at 1699 ng/L six hours after. Pertinent labs were as follows: calcium 8.9 mg/dL, D-dimer 0.35 mg/L, ferritin 348.4 ng/L, LDH 263 U/L, and CRP 51.4 mg/L. Real-time RT-PCR COVID-19 test (DiaSorin Molecular) was positive. No significant electrolyte or other lab abnormalities were noted. Echocardiogram was performed and showed a new onset of reduced ejection fraction between 30 and 35%. He did not have a known history of reduced ejection fraction. His oxygen requirement was 2 L on nasal cannula saturating between 93 and 100%. He was moved to the CCU for further evaluation and management.

The patient was started on a low-dose dopamine infusion at 10 mcg/kg/min and dexamethasone 6 mg IV QD. Infectious disease team was consulted, and remdesivir was started based on a multidisciplinary team approach. Blood pressure remained between 140/76 and 162/62. The patient remained awake and comfortable. The patient was offered both cardiac catheterization and permanent pacemaker placement; however, after an extensive discussion with the patient and his son, the patient refused all invasive therapeutic intervention. The patient was discontinued off the dopamine infusion as well as transcutaneous pacing; however, he was continued on methylprednisolone and remdesivir. On posthospitalization day 6, his symptoms at onset had grossly improved, and given his aversion to testing, no follow-up imaging was conducted. He was discharged safely still in AV block with home oxygen.

## 3. Discussion

Complete AV block etiology is exhaustive including systemic diseases, ischemic heart disease, myocarditis, structural heart disease, and infectious disease. It has been reported in Takotsubo cardiomyopathy as well [5]. Arrhythmias and cardiovascular manifestation, including myocarditis and Takotsubo cardiomyopathy, has been seen in COVID-19 infections in approximately 17% hospitalized patients and 44% ICU patients. It may be attributed to multiple factors including hypoxia, electrolyte derangement, and cytokine surge [6].

Although this patient's etiology of his complete AV block may result from multiple factors, given the acuity in setting of concurrent COVID-19 infections, top differentials include viral myocarditis, COVID-19-induced Takotsubo cardiomyopathy complicated by a complete AV-block, or a direct conduction pathway infection. Ischemia cannot be completely ruled out as well. Due to his distant history, the patient did not know whether his right coronary artery was stented or diseased. No prior catheterization report was available as most recent catheterization was 16 years prior out-of-state. COVID-19 has been documented to infect cells via the angiotensin-converting enzyme-2 (ACE-2) receptor [7]. Therefore, a direct infection of the conduction pathway is plausible. Complete AV blocks are also clinical symptoms of myocarditis. Otherwise, vaccine-induced myocarditis complications should also be considered [8].

Management is complicated given the mortality rate of COVID-19. There has been a case report of transient high-grade AV blocks in COVID-19 infected patients [4]. Whether all patients with COVID-19 induced high-grade AV blocks require a pacemaker remains a question. Furthermore, in patients with increased probability of mortality, discussion should revolve around risks and benefits as well as quality of life even after a pacemaker placement. Palliative service consultation should also be considered. If the patient is stable with a seemingly irreversible complete AV block, a pacemaker should be recommended.

Throughout the pandemic, multiple medications have been investigated for treatment options. In particular, remdesivir has shown to decrease time to recovery in hospitalized patients as well as the rate of lower respiratory tract infection [9]. However, remdesivir has been associated with duration-dependent cardiac adverse effects due to mitochondrial toxicity and similarities of its active metabolite to adenosine triphosphate. These effects include SA node automaticity reduction, AV nodal abnormalities, QRS prolongation, and myocyte toxicity [10]. The discussion to administer remdesivir in COVID-19 patients with high-degree AV block should be approached in a multidisciplinary fashion.

With the rise of the new COVID-19 variants, vaccination and booster doses should be recommended for all patients without contraindication. It is possible that the virus may mutate to present with more cardiologic pathologies given the pathophysiology. Depending on the type of vaccine, individuals may benefit with protection from variants [11].

## 4. Conclusion

We present a case of a Pfizer-vaccinated COVID-19 infected individual whose initial presenting symptoms included a complete AV block. To the author's knowledge, this is the first documentation of such case.
